# Viral-mediated oncolysis is the most critical factor in the late-phase of the tumor regression process upon vaccinia virus infection

**DOI:** 10.1186/1471-2407-11-68

**Published:** 2011-02-14

**Authors:** Stephanie Weibel, Viktoria Raab, Yong A Yu, Andrea Worschech, Ena Wang, Francesco M Marincola, Aladar A Szalay

**Affiliations:** 1 Department of Biochemistry, Biocenter, University of Wuerzburg, D-97074 Wuerzburg, Germany; 2 Rudolf Virchow Center, Research Center for Experimental Biomedicine, University of Wuerzburg, D-97078 Wuerzburg, Germany; 3 Institute for Molecular Infection Biology, University of Wuerzburg, D-97078 Wuerzburg, Germany; 4 Genelux Corporation, San Diego Science Center, San Diego, CA 92109, USA; 5 Infectious Disease and Immunogenetics Section, Department of Transfusion Medicine, Clinical Center, National Institutes of Health, Bethesda, MD 20892, USA

## Abstract

**Background:**

In principle, the elimination of malignancies by oncolytic virotherapy could proceed by different mechanisms - e.g. tumor cell specific oncolysis, destruction of the tumor vasculature or an anti-tumoral immunological response. In this study, we analyzed the contribution of these factors to elucidate the responsible mechanism for regression of human breast tumor xenografts upon colonization with an attenuated vaccinia virus (VACV).

**Methods:**

Breast tumor xenografts were analyzed 6 weeks post VACV infection (p.i.; regression phase) by immunohistochemistry and mouse-specific expression arrays. Viral-mediated oncolysis was determined by tumor growth analysis combined with microscopic studies of intratumoral virus distribution. The tumor vasculature was morphologically characterized by diameter and density measurements and vessel functionality was analyzed by lectin perfusion and extravasation studies. Immunological aspects of viral-mediated tumor regression were studied in either immune-deficient mouse strains (T-, B-, NK-cell-deficient) or upon cyclophosphamide-induced immunosuppression (MHCII^+^-cell depletion) in nude mice.

**Results:**

Late stage VACV-infected breast tumors showed extensive necrosis, which was highly specific to cancer cells. The tumor vasculature in infected tumor areas remained functional and the endothelial cells were not infected. However, viral colonization triggers hyperpermeability and dilatation of the tumor vessels, which resembled the activated endothelium in wounded tissue. Moreover, we demonstrated an increased expression of genes involved in leukocyte-endothelial cell interaction in VACV-infected tumors, which orchestrate perivascular inflammatory cell infiltration. The immunohistochemical analysis of infected tumors displayed intense infiltration of MHCII-positive cells and colocalization of tumor vessels with MHCII^+^/CD31^+ ^vascular leukocytes. However, GI-101A tumor growth analysis upon VACV-infection in either immunosuppressed nude mice (MHCII^+^-cell depleted) or in immune-deficient mouse strains (T-, B-, NK-cell-deficient) revealed that neither MHCII-positive immune cells nor T-, B-, or NK cells contributed significantly to VACV-mediated tumor regression. In contrast, tumors of immunosuppressed mice showed enhanced viral spreading and tumor necrosis.

**Conclusions:**

Taken together, these results indicate that VACV-mediated oncolysis is the primary mechanism of tumor shrinkage in the late regression phase. Neither the destruction of the tumor vasculature nor the massive VACV-mediated intratumoral inflammation was a prerequisite for tumor regression. We propose that approaches to enhance viral replication and spread within the tumor microenvironment should improve therapeutical outcome.

## Background

During the past several years, many reports have confirmed that intratumoral as well as systemic delivery of a variety of virus strains leads to viral replication in tumors accompanied by oncolysis of tumor cells [1-3]. Most of these replicating oncolytic viruses specifically target solid tumors [4], which is a significant advantage over the use of conventional chemo- and radiotherapy. Although oncolytic viruses are successfully used as tumor-targeting agents in animal models, the modulation of the tumor microenvironment by the viruses as well as the virus-host interaction dynamics are not well understood and therefore, the exact underlying mechanism leading to tumor elimination is less clear [5-8].

Malignant tumors are complex organ-like tissues composed of ever-evolving neoplastic cells and non-neoplastic cellular components, including fibroblasts, endothelial cells and immune cells, surrounded by an extracellular matrix [9]. These stromal components have an important function in maintaining and supporting solid tumor growth and viral infection could theoretically interfere with all of them. Moreover, viruses induce local inflammation at sites of infection leading to local remodeling of the infected tissue such as activation of the vasculature and local recruitment of immune cells. Up to date, the long-term VACV-infected tumor microenvironment is not described in the literature and the mechanism of VACV-mediated tumor regression is less clear. Theoretically, three possible mechanisms may explain virus-mediated tumor elimination - tumor cell specific oncolysis [10], destruction of the tumor vasculature [11,12] followed by oxygen and nutrients deprivation, an anti-tumoral immune response [7,13], or a combination of these mechanisms [14,15]. For optimization of oncolytic virus therapy it is desired to determine which factors contribute to most optimal virus-mediated tumor regression.

Recently, Zhang et al. [16,17] have introduced a novel attenuated recombinant vaccinia virus GLV-1h68 and described its improved safety profile in comparison to the parental wild-type LIVP strain. Furthermore, they documented the successful application as an oncolytic agent in therapy of human breast tumor xenografts in nude mice.

In this study, we used the GLV-1h68 vaccinia virus strain to investigate the factors that may contribute to VACV-mediated tumor regression, with the final aim of improving therapeutic outcomes. We found that GLV-1h68 infection of GI-101A human breast tumor xenografts in nude mice leads to specific oncolytic destruction of the tumor tissue accompanied by tumor shrinkage. Interestingly, endothelial cells were uninfected and the vasculature remained functional. However, the tumor vasculature in infected areas strongly resembled the activated endothelium in wounded tissue, characterized by vessel dilatation, hyperpermeability and the increased expression of adhesion molecules. Furthermore, viral infection triggered increased expression of genes involved in leukocytes recruitment in the late regression phase leading to massive MHCII-positive leukocytes infiltration via the activated tumor vasculature. However, immunosuppression (MHCII^+^-cell depletion) of tumor-bearing, VACV-infected animals as well as the use of T-, B-, and NK-deficient mouse models for tumor growth analysis revealed that none of these immune cells are a prerequiste for VACV-mediated GI-101A tumor regression. Our results suggested that viral oncolysis is the critical factor for tumor elimination in the late regression phase mediated by VACV. We therefore propose that the most beneficial way to improve therapeutic outcomes with the oncolytic vaccinia virus GLV-1h68 strain is to enhance viral replication and spread within the tumor tissue.

## Methods

### Cell lines

GI-101A human ductual breast adenocarcinoma cells were kindly provided by A. Aller (Rumbaugh-Goodwin Institute for Cancer Research, Inc., FL, USA) and cultured in RPMI 1640 supplemented with 5 ng/ml β-estradiol and 5 ng/ml progesterone (Sigma Aldrich, Taufkirchen, Germany), 1 mM sodium pyruvate, 10 mM HEPES, 20% FBS, 100 Units/ml penicillin, and 100 μg/ml streptomycin (PAA Laboratories, Cölbe, Germany). African green monkey kidney fibroblasts (CV-1) were obtained from the American Type Culture Collection (ATCC-No. CCL-70) and cultured in DMEM supplemented with 10% FBS. The murine endothelial cell line 2H-11 (ATCC-No. CRL-2163) as well as mouse brain endotheliomas bEnd.3 (kindley provided by G. J. Hämmerling, Deutsches Krebsforschungszentrum, Heidelberg, Germany) were obtained in DMEM with 10% FBS. Human umbilical vein endothelial cells (HUVEC) were obtained from PromoCell (Heidelberg, Germany) and cultured in M199 medium supplemented with 10% FBS, 10 ng/ml human EGF and 50 μg/ml endothelial cell growth supplement (Sigma Aldrich). The human kidney cell line 293FT was obtained from Invitrogen GmbH (Karlsruhe, Germany) and cultured in DMEM supplemented with 10% FBS, 0.1 mM non-essential amino acids, 6 mM L-glutamine, and 1 mM sodium pyruvat. All cells were maintained at 37°C and 5% CO_2_.

### Viruses and plasmids

The construction of the attenuated vaccinia virus strain GLV-1h68 was described previously by Zhang et al. [16]. Briefly, three expression cassettes (encoding for *Renilla *luciferase-GFP fusion protein, β-galactosidase and β-glucuronidase) were recombined into the *F14.5L*, *J2R *and *A56R *loci, respectively, of the LIVP strain viral genome. Viruses were propagated in CV-1 cells and purified through sucrose gradients.

The RFP-expressing GI-101A cell line was constructed using the ViraPower™ Gateway Cloning and Lentiviral Expression System Kit (Invitrogen GmbH, Germany) in accordance with the manufacture's instructions. The *mRFP*-encoding plasmid pCR-TK-Sel-*mRFP *was provided by Q. Zhang (Genelux Corporation, San Diego) and used as a template for PCR amplification of the *mRFP *gene using primers containing attB recombination sites for gateway cloning (forward-*attB1-mRFP*: 5'-GGGGACAAGTTTGTACAAAAAAGCAGGCTGCCACCATGGCCTCCTCCGAGG-3', reverse-*attB2-mRFP*: 5'-GGGGACCACTTTGTACAAGAAAGCTGGGTCAGAATTCGCCCTTTCATTAGG-3'). The *mRFP*-containing lentiviral vectors were generated by gateway recombination between the pDONR-221-*mRFP *entry vector and the pLenti6/V5-DEST destination vector. The *mRFP*-containing replication-incompetent Lentiviruses for transduction of GI-101A cells were produced in 293FT cells using Lipofectamine™2000 for transfection with the ViraPower™ Packaging Mix and the pLenti6/V5-DEST-*mRFP *expression plasmid. Stable-expressing GI-101A-RFP clones were selected using 10 μg/ml blasticidin.

### Tumor inoculation and administration of the virus

All animal experiments were carried out in accordance with protocols approved by the Regierung von Unterfranken, Germany (permit number: 55.2-2531.01-17/08).

Six-week-old female athymic nude *Foxn1*^*nu *^mice were obtained from Harlan Winkelmann GmbH (Borchen, Germany). Six-week-old female B6.12956-*Rag2*^*tm1Fwa *^N12 mice and Tac:NIHS-*Lyst*^*bg*^*Foxn1*^*nu*^*Btk*^*xld *^mice were ordered from Taconic Inc. (Hudson, NY, USA). GI-101A breast cancer cells (5 × 10^6^/100 μl PBS) were subcutaneously (s.c.) injected into the abdominal right flank and tumor volume was calculated as (length × width^2^)/2. For all experiments, tumors were grown up to 200-400 mm^3 ^in size (4-6 weeks) before viral administration. A single viral dose of 1 × 10^6 ^or 5 × 10^6 ^plaque forming units (p.f.u.) in 100 μl PBS was injected either intraveneously (i.v.) via the tail vein or via the retro-orbital (r.o.) sinus vein. For r.o. injection, animals were anesthetized using 75 mg/kg ketamine (Pfizer, Karlsruhe, Germany) and 20 mg/kg xylazine (Bayer, Leverkusen, Germany).

### Immunohistochemistry

For histological studies, tumors were excised and snap-frozen in liquid N_2_, followed by fixation in 4% paraformaldehyde/PBS pH 7.4 for 16 h at 4°C. Fixed tumors were rinsed in PBS and embedded in 5% (w/v) low-melt agarose (AppliChem, Darmstadt, Germany). Tissue-sectioning (100 μm) was performed using the Leica VT1000S Vibratome (Leica, Heerbrugg, Switzerland) and the labelling procedures were previously described in detail elsewhere [18].

### Fluorescence microscopy

The fluorescence-labelled preparations were examined using the MZ16 FA Stereo-Fluorescence microscope (Leica) equipped with the digital DC500 CCD camera and the Leica IM1000 4.0 software (1300 × 1030 pixel RGB-color images) as well as the Leica TCS SP2 AOBS confocal laser microscope equipped with an argon, helium-neon and UV laser and the LCS 2.16 software (1024 × 1024 pixel RGB-color images). Digital images were processed with Photoshop 7.0 (Adobe Systems, Mountain View, CA) and merged to yield overlay images.

### Fluorescence intensity measurements

Fluorescence intensity of the CD31- and MHCII-labelling in 100-μm-thick Vibratome sections of control tumors and infected areas of GLV-1h68-colonized tumors was measured on digital images (× 50 objective, × 1 ocular, tissue region 2700 μm by 2150 μm) of specimens stained for CD31 or MHCII immunoreactivity. On the fluorescence microscope, the background fluorescence was set to a barely detectable level by adjusting the gain of the CCD camera before all the images were captured with identical settings. RGB-images were converted into 8-bit gray scale images (intensity range 0 - 255) using Photoshop 7.0. The fluorescence intensity of the CD31-labelling represented the average brightness of all vessel-related pixels and was measured using Image J software http://rsbweb.nih.gov/ij. For CD31-labelling the mean value was calculated for nine images (three images of three different control and GLV-1h68-infected tumors) and presented with standard deviation.

The extent of the viral distribution in GLV-1h68-colonized tumors was measured by the GFP fluorescence signal on digital images (× 10 objective, × 1 ocular, image size 14 mm by 11.1 mm) of two whole tumor cross-sections (100 μm) of five or six different tumors. The whole area of the tumor cross-section was determined by Hoechst-labelling of cell nuclei. Both, GFP and Hoechst fluorescence images were converted into 8-bit gray scale images (intensity range 0 - 255) using Photoshop 7.0. The background fluorescence of GFP images was set to the fluorescence intensity of < 20 using Image J software. A fluorescence intensity of 20 was thus established as the threshold for distinguishing pixels of the GFP signal from those of the background. The area of pixels (inch^2^) on GFP images (fluorescence intensity > 20) as well as on Hoechst images (fluorescence intensity > 0) was measured by Image J and the proportion of infected tissue was calculated for two images from each tumors (n = 6). Mean values + standard deviations are shown.

### Measurements of microvessel density and vessel diameter

The vascular density was determined in microscopic images (× 200 objective, × 1 ocular, tissue region 680 μm by 540 μm) of CD31-labelled tumor sections. On the fluorescence microscope, for each image the CD31 fluorescence was set to a clearly detectable level by individually adjusting the gain of the CCD camera before the images were captured. All images were decorated with five horizontal lines at identical positions using Photoshop 7.0 and all vessels which intersected these lines were counted to yield the vascular density. The vascular density was calculated for nine images (three images of three different control and GLV-1h68-infected tumors) and presented as mean values with standard deviations.

The vessel diameter was measured on digital images (× 200 objective, × 1 ocular) of CD31-labelled 100-μm-thick tumor cross-sections using Leica IM1000 4.0 software. Images of control and infected tumors (GLV-1h68-infected area) were obtained with individual exposure times to get optimal CD31 signals and exclude signal-dependent variability of vessel diameter. Seven horizontal lines were drawn across each image and the diameter of all blood vessels that intersected these lines was measured (5 images per tumor). Mean values + standard deviations are shown.

### Antibodies, reagents and treatment of animals

Endothelial cells were labelled with monoclonal rat anti-mouse CD31 antibody (BD PharMingen, San Diego, CA) or hamster anti-mouse CD31 antibody (Chemicon, International, Temecula, CA). Pericytes were labelled with Cy3-conjugated monoclonal mouse anti-mouse α-smooth muscle actin (SMA) (Sigma Aldrich). Basement membrane was labeled using polyclonal rabbit anti-mouse collagen IV antibody (Abcam, Cambridge, UK). Immune cells were labeled using rat anti-mouse MHCII antibody (B, dendritic cells, monocytes, macrophages) and rat-anti mouse CD45 antibody (common leukocyte antigen) (eBioscience, San Diego, CA).

The Cy3- or Cy5-conjugated secondary antibodies (donkey) were obtained from Jackson ImmunoResearch (West Grove, PA).

Phalloidin-TRITC (Sigma Aldrich) was used to label actin and Hoechst 33342 to label nuclei in tissue sections.

For the labelling of functional blood vessels in tumors, mice were anesthetized using 75 mg/kg ketamine and 20 mg/kg xylazine, followed by the injection of 100 μg of biotinylated-*Lycopersicum esculentum *lectin (Vector Laboratories, Burlingame, CA) via the tail vein of the mice. Two minutes later the chest was opened, and the vasculature was perfused at a pressure of 120 mmHg with fixative (4% paraformaldehyde/PBS pH 7.4) from a cannula inserted into the left ventricle. After fixation, tumors were removed and prepared for histology. Tumor cross-sections (100 μm) were labelled with Cy3-conjugated streptavidin (Sigma Aldrich) to visualize the lectin-labelled tumor vasculature.

Nonspecific rat-IgG from Jackson ImmunoResearch was used in extravasation studies and injected intravenously into tumor-bearing mice (11 mg/kg body weight). After 6 h incubation, the treated tumors were excised and used for histological analysis. Surface plot profiles of the IgG extravasation pattern were prepared using ImageJ software.

For immunosuppression a stock solution of cyclophosphamide monohydrate (42 mg/ml) (Sigma Aldrich) was prepared in water and sterile filtered. Immediately before use, the stock solution was diluted 1:1 in 1.8% NaCl to yield a final concentration of 21 mg/ml. CPA was administered by intraperitoneal injection twice per week throughout the entire duration of the study. The treatment was started 10 days p.i. with an initial dose of 140 mg/kg body weight followed by 100 mg/kg body weight. The dose and schedule was based on previously published studies of CPA immunosuppression in mice and hamsters [19].

### Viral replication *in vitro*

For viral replication assays, tumor cells as well as endothelial cells were seeded in triplicates into 24-well plates to reach a confluency of 80% after a culture period of 12-16 h. Before infection, cell layers were starved with individual starvation media containing 1% FBS for 24 h and were finally infected with GLV-1h68 at m.o.i. of 0.01. After 1 h of incubation, the infection medium was replaced by fresh starvation medium and cells were cultured for further 6, 24 and 48 h, respectively. At the indicated time points, cells and supernatants were harvested and after three thaw-freeze cycles, serial dilutions of the lysates were titered by standard plaque assays on CV-1 cells.

### Co-culture experiments

To mimic *in vivo *conditions, we cultured endothelial cells on growth factor reduced Matrigel Matrix (BD Biosciences, Heidelberg, Germany), which is a soluble basement membrane extract. For co-culture experiments, we coated 24 well plates with 100 μl of Matrigel for 30 min at 37°C. Endothelial cells (1 × 10^4 ^cells/well) were seeded into 24 well plates and allowed to assemble into tube-like structures. Three hours later GI-101A-RFP tumor cells were seeded into these wells and co-cultures were incubated for 12-15 h. Co-cultures were infected with GLV-1h68 at m.o.i. of 0.5 for 1 h, before the medium was replaced with virus-free medium. The degree of infection was microscopically determined after 24 h.

### Microarray performance and statistical analysis

Total RNA from both infected and uninfected GI-101A xenografts at days 21 and 42 post VACV infection was extracted using Trizol reagent (Sigma Aldrich) according to the manufacturer's instructions. Total RNA was amplified into anti-sense RNA (aRNA) as previously described [20,21] and the quality of both, total RNA and secondarily amplified RNA was tested with the Agilent Bioanalyzer 2000 (Agilent Technologies, Palo Alto, CA). Confidence about array quality was based on the principle of reference concordance as previously described [22]. Mouse reference RNA was prepared by homogenization of the following mouse tissues (lung, heart, muscle, kidneys and spleen) and RNA was pooled from 4 mice. Pooled reference and test aRNA was isolated and amplified in identical conditions during the same amplification/hybridization procedure to avoid possible inter-experimental biases. Both, reference and test aRNA was directly labeled using ULS aRNA Fluorescent labeling Kit (Kreatech, Netherlands) with Cy3 for reference and Cy5 for test samples.

Whole genome mouse 36 k oligo arrays were printed in the Infectious Disease and Immunogenetics Section of the Department of Transfusion Medicine (IDIS), Clinical Center, National Institute of Health, Bethesda using oligos purchased from Operon (Huntsville, AL). The Operon Array-Ready Oligo Set (AROS™) V 4.0 contains 35,852 longmer probes representing 25,000 genes and about 38,000 gene transcripts and also includes 380 controls. The design is based on the Ensembl Mouse Database release 26.33b.1, Mouse Genome Sequencing Project, NCBI RefSeq, Riken full-length cDNA clone sequence, and other GenBank sequence. The microarray is composed of 48 blocks and one spot is printed per probe per slide. Hybridization was carried out in a water bath at 42°C for 18-24 hours and the arrays were then washed and scanned on a Gene Pix 4000 scanner at variable PMT to obtain optimized signal intensities with minimum (< 1% spots) intensity saturation.

Resulting data files were uploaded to the mAdb databank http://nciarray.nci.nih.gov and further analyzed using BRBArrayTools developed by the Biometric Research Branch, National Cancer Institute [23]http://linus.nci.nih.gov/BRB-ArrayTools.html and Cluster and Treeview software [24]. The global gene-expression profiling consisted of 16 experimental samples. Global expression data were filtered using automated filtering option of BRBArray software. Therefore, genes involved in pathways such as "adhesion molecules on lymphocytes, B lymphocytes cell surface molecule, cytokines and inflammatory response, monocytes and its surface molecules, neutrophiles and its surface molecules, T cytotoxic cell surface molecules, T helper cell cytotoxic molecules" as listed by the Biocarta database were included. Genes that belonged to at least one of those pathways, that were present in more than 10 experimental samples (≥ 60%) and with a fold change of two in at least one sample passed the filter. Gene ratios were average corrected across experimental samples. Subsequent cluster analysis applying uncentered correlation algorithm with genes involved in selected pathways as listed above allowed experimental samples to cluster according to their biological similarity. Treeview program was used for visualization of array data [25].

### Statistics

A two-tailed Student's *t *test was used for statistical analysis. *P *values of < 0.05 were considered statistically significant.

## Results

### GLV-1h68 colonization of human breast tumor xenografts in nude mice leads to specific oncolytic destruction of the tumor tissue

**Figure 1:**
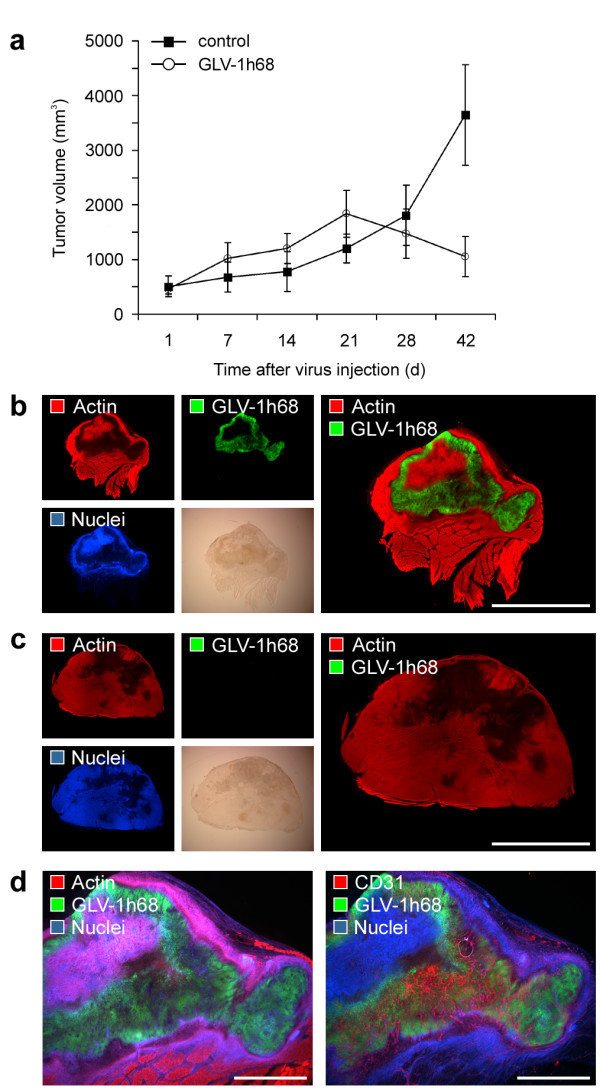
**Oncolytic destruction of human GI-101A breast tumor xenografts**. GI-101A tumor-bearing mice were intraveneously (i.v.) injected with 5 × 10^6 ^pfu/100 μl PBS GLV-1h68 or PBS as a control. (a) Tumor growth was monitored weekly by measuring the tumor volume of six mice in each group. Shown are the mean values +/- standard deviations. The study was repeated in three independent experiments. (b, c) Whole tumor cross-sections (100 μm) of GLV-1h68-infected GI-101A (b) and control tumors (c) 42 days p.i. were stained with Phalloidin-TRITC (red) to label the actin cytoskeleton and Hoechst 33342 (blue) to visualize cellular nuclei; GFP fluorescence (green) indicated viral-infected cells. (d) Serial sections of whole tumor cross-sections of 42-days-colonized GI-101A tumors were labelled either with Phalloidin-TRITC (actin) or anti-CD31 antibody (red). In both sections nuclei were stained with Hoechst (blue) and GLV-1h68-infected cells were indicated by GFP fluorescence (green). The colonized, necrotic tumor tissue showed a dense CD31-positive vascular network. All images are representative examples. Scale bars represent 5 mm (b, c), 2 mm (d).

We have previously reported that viral colonization of human breast tumor xenografts in nude mice leads to a remarkable tumor regression over time following a characteristic three-phase growth pattern [16]. After intraveneous injection (i.v.) of 5 × 10^6 ^pfu (plaque forming unit) into tumor-bearing mice, GI-101A breast tumors started to increase in volume during the first three weeks (Phase I, early phase) reaching a peak volume at about day 21 (Phase II), and begin then to decline in size continuously (Phase III, regression phase) (Figure 1a). The volume of colonized tumors about 6 weeks after virus treatment is only 31% of that of untreated tumors.

To investigate the localization of viral particles within colonized tumor xenografts, we analyzed the distribution of VACV-infected, GFP-expressing cells in histologically prepared whole tumor cross-sections. As shown in Figure 1b and Additional file 1: Figure 1a, 42 days post infection (p.i.) a large part (55.25 +/- 7.26%) of the tumor tissue showed GFP fluorescence indicating extensive viral distribution. The comparison of GI-101A control tumors with colonized GI-101A tumors of equivalent age revealed that viral colonization leads to extensive necrotic tissue destruction as displayed by the lack of actin cytoskeleton and cell nuclei labelling (Figure 1b,c). Therefore, much of the tumor mass that remained and was being measured in GLV-1h68-colonized tumors was actually necrotic debris and calcification. In contrast, at earlier time points (7 and 21 days p.i.) a more restricted patch-like distribution pattern of GFP fluorescence and necrotic tissue destruction was observed, revealing the replication- and dissemination-characteristics of vaccinia virus GLV-1h68 strain (Additional file 1: Figure 1b). The tumor tissue-specific nature of the oncolytic activity of GLV-1h68 was further supported by the fact that viral infection was restricted to RFP-expressing tumor cells. Consequently, the ongoing infection leads to a decline of RFP fluorescence in GI-101A-RFP tumors indicating tumor cell-specific necrosis (Additional file 1: Figure 1c). Interestingly, the infected, necrotic tumor tissue at day 42 p.i. was highly vascularized demonstrated by positive CD31-labelling of the tumor vasculature in colonized tumor areas (Figure 1d). This finding supported the specific oncolytic character of viral-mediated tumor tissue destruction and eliminated the notion that a loss of blood supply in the infected area could be the reason for tissue necrosis.

### GLV-1h68 does not infect the endothelial cells of the tumor vasculature

The tumor vasculature is an important part of the tumor microenvironment which supports tumor growth by delivery of nutrients, oxygen and immune cells. Therefore, the destruction of the vascular network in tumors offers one hypothetical therapeutic strategy for destruction of the tumor mass [26].

**Figure 2:**
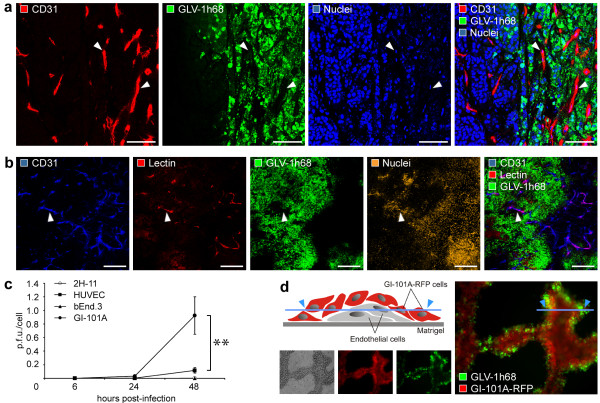
**GLV-1h68 does not infect endothelial cells**. (a) Confocal image showing the morphological intact tumor vasculature (arrowheads) in the GLV-1h68-infected (i.v.) GI-101A tumor tissue 42 days p.i.; the tumor vasculature was labelled with anti-CD31 antibody (red), GLV-1h68-infection was indicated by GFP (green), and nuclei were stained with Hoechst (blue). (b) Systemic perfusion of the vasculature with lectin revealed colocalization (arrowhead) of CD31-positive tumor blood vessels (blue) and biotinylated-*Lycopersicum esculentum *lectin (red). (c) Viral replication in GI-101A tumor cells, human endothelial (HUVEC) and murine endothelial (2H-11, bEnd.3) cells inoculated with GLV-1h68 at m.o.i. of 0.01. Infected cells were harvested 6, 24, and 48 h after infection. Shown are viral titers of cell monolayers as p.f.u./cell in triplicate samples +/- standard deviations determined by plaque assay. (d) Infection of co-cultures of GI-101A-RFP (red) and HUVEC cells (unlabelled) on Matrigel matrix with GLV-1h68 (green) with m.o.i. 0.5 confirmed the specific infection of tumor cells. The illustration visualized the formation of tube-like structures composed of endothelial cells surrounded by tumor cells on Matrigel matrices. The asterisk (c) indicate a significant difference between experimental groups (** *P *< 0.01; Student's *t *test). All images are representative examples. Scale bars represent 75 μm (a) and 300 μm (b).

To assess the impact of viral tumor colonization on the tumor vasculature, we analyzed the CD31-positive vascular network in tissue sections by confocal microscopy. As shown in Figure 2a, CD31-positive blood vessels in the infected tumor areas of GI-101A tumors 42 days p.i. are morphologically intact and are not infected by VACV as indicated by the lack of the colocalization of CD31-labelling and that of GFP fluorescence. These findings strongly indicate that VACV possesses an inherent replication-specificity for tumor cells and destruction of the tumor vasculature is not a prerequisite of the GLV-1h68-mediated tumor elimination.

To analyze whether blood vessels in GLV-1h68-infected tumor areas are still functional, we intravenously injected biotinylated-*Lycopersicum esculentum *lectin which specifically labels perfused blood vessels. Indeed, we could demonstrate that the tumor vasculature in the GLV-1h68-colonized areas was perfused and therefore still connected to the peripheral blood stream (Figure 2b).

To confirm the cell specificity, we carried out cell culture infection experiments by comparing viral infectivity and replication in GI-101A tumor cells and in endothelial cells. We compared three different endothelial cell lines, two murine cell lines (2H-11, bEnd.3) as well as one human cell line (HUVEC), to exclude species-specific endothelial cell differences, which could affect viral infection. Plaque assays of VACV-infected cell cultures revealed that highest viral infection and replication occured in the GI-101A tumor cell line and viral particles replicated in endothelial cell lines regardless to the origin significantly lesser (Figure 2c). To more clearly demonstrate tumor cell specificity of this virus and also to mimic the tumor infection, we co-cultured GI-101A-RFP tumor cells and endothelial HUVEC cells on Matrigel matrices. The extracellular matrix extract lead to the formation of tube-like structures composed of endothelial cells surrounded by tumor cells (Figure 2d). The results demonstrated that only the surrounding tumor cells were infected by VACV as visualized by GFP fluorescence and the internal endothelial cells remained uninfected (Figure 2d). Similarly, co-culturing of murine 2H11 endothelial cells with GI-101A tumor cells on Matrigel matrix also showed tumor cell-specific VACV replication only (data not shown).

### Tumor colonization by GLV-1h68 does not stimulate neo-angiogenesis but upregulates CD31 expression in endothelial cells and genes involved in leukocytes recruitment

**Figure 3:**
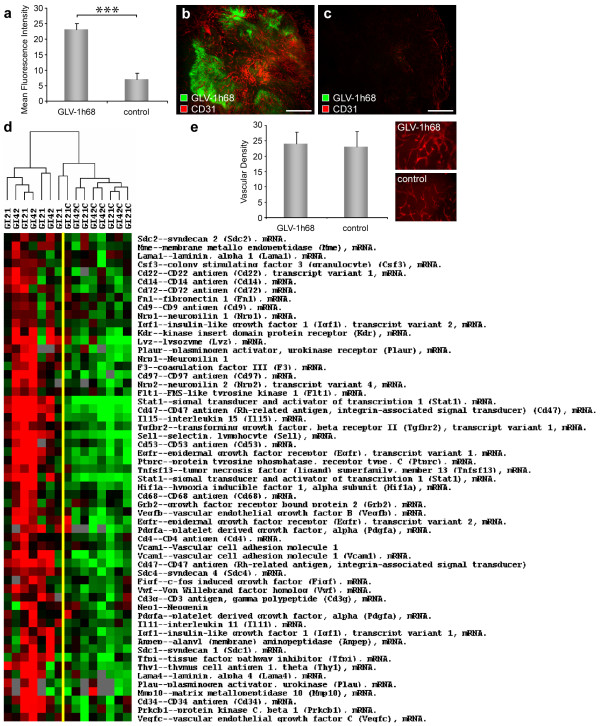
**Viral tumor infection activates the tumor vasculature**. Athymic nude mice bearing human GI-101A tumors were injected with 5 × 10^6 ^pfu of GLV-1h68 and tumors were harvested 42 days p.i.. (a-c) GLV-1h68-colonized GI-101A tumors showed increased CD31 fluorescence intensity compared to GI-101A control tumors. The tumor vasculature was labelled with anti-CD31 antibody (red) and the fluorescence signals in 9 images (x50 magnification) were measured using ImageJ. Shown are the mean values +/- standard deviations. (e) GLV-1h68-infected and control GI-101A tumors showed the same level of vascularization. Vascular density was measured in CD31-labelled tumor cross-sections (nine per group). Shown are the mean values +/- standard deviations. (d) Gene expression profile of both, VACV infected and uninfected GI-101A xenografts 21 and 42 days p.i.. Genes were selected based on pathway analysis as described in material and methods and clustered using uncentered correlation algorithm. Genes that passed the filtering criteria (at least 2fold change in one experiment and 60% presence call across the experimental set) are displayed. The asterisk (a) indicate a significant difference between experimental groups (*** *P *< 0.001; Student's *t *test). All images are representative examples. Scale bars represent 500 μm (a, b).

To address the influence of viral tumor colonization on the tumor vasculature in GI-101A tumors, we analyzed first the intensity of CD31-labelling in 42-days GLV-1h68-infected and control GI-101A tumors. Fluorescence intensity of the CD31 signal of blood vessels was measured in microscopic images in immunohistochemically stained sections of GI-101A tumors. The results revealed that the number of vessel-related pixels in infected tumor areas increased more than 3-fold in comparison to non-injected control tumors of same age (Figure 3a-c).

To determine whether the increase in the CD31 immunofluorescence signal in infected tumors resulted from an increased vascular density or is due to an up-regulation of CD31 expression, we analyzed CD31-labelled cross sections of control and GLV-1h68-colonized tumors in a microscopic study. We selected individual exposure times to visualize the CD31-labelled vasculature in each section independent of the CD31 expression level. Individual exposure times clearly revealed that there was no quantitative difference in the vascular density, thus confirmed the viral-infection-induced upregulation of CD31 in the vasculature of colonized tumors (Figure 3d).

Since tumor colonization with GLV-1h68 lead to an up-regulation of CD31, which mediate transendothelial migration of immune cells to sites of infection, we analyzed the expression profile of genes involved in leukocytes recruitment in VACV-infected GI-101A tumors versus uninfected control tumors using a custom-made, whole genome 36 K mouse array platform. In general, the analyzed tumor samples showed no characteristic pro-angiogenic signature, but revealed an significant up-regulation of genes (e.g. *Stat1*, *Sell*, *Vcam1, Itgb2, F11r, Jam3*, *Cd47*, *Cd97*, *Sdc1*, *Sdc2*, *Sdc4*) involved in leukocyte-endothelial cell interaction and recruitment of immune cells to sites of infection (Figure 3e).

In summary, VACV-infection of tumor xenografts in nude mice did not change the vascular density of the tumor tissue, but altered significantly the expression profile of tumor-derived endothelial and inflammatory cells, which may have resulted in an increased recruitment and migration of immune cells to the infected tumor tissue.

### Oncolytic virus infection triggers intratumoral blood vessel dilatation and increased vascular permeability

**Figure 4:**
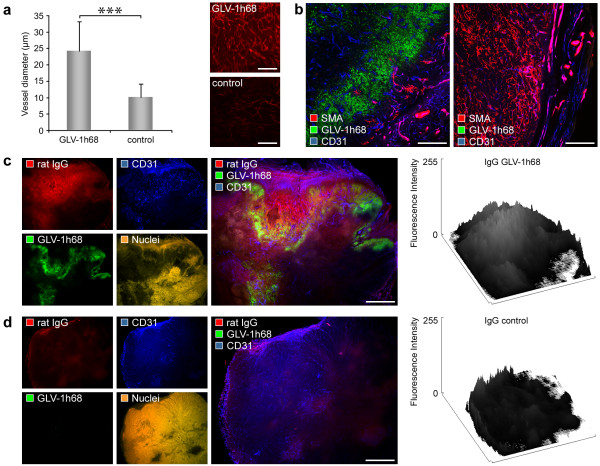
**GLV-1h68 colonization of GI-101A tumors leads to blood vessel dilatation, destruction of pericytes, and increased vessel permeability**. Athymic nude mice bearing human GI-101A tumors were retro-orbitally (r.o.) injected with 5 × 10^6 ^pfu of GLV-1h68 and tumors were harvested 42 days p.i.. (a) GLV-1h68-colonized GI-101A tumors showed increased vessel size (mean diameter 24.17 +/- 9.07 μm) compared to GI-101A control tumors (10.09 +/- 4.01 μm). The tumor vasculature was labelled with anti-CD31 antibody (red) and the diameter of all blood vessels (12-18 vessels/image) in 5 images (× 200 magnification) was measured using Leica IM1000 4.0 software. Shown are the mean values +/- standard deviations. (b) Confocal images showed destruction of pericytes in GLV-1h68-infected GI-101A tumors. Pericytes were labelled using Cy3-conjugated anti-SMA antibody (red), tumor vasculature was labelled with anti-CD31 antibody (blue), and viral infection was indicated by GFP fluorescence (green). (c, d) Extravasation of unspecific rat IgGs in 42-days-colonized (c) and control GI-101A tumors (d); mice were injected with 11 mg/kg rat IgGs 6 h before the tumors were fixed, histologically prepared and labelled with Cy3-conjugated anti-rat antibody to visualize extravasated IgGs (red). The tumor vasculature was labelled with anti-CD31 antibody (blue), GLV-1h68-infection was indicated by GFP (green), and nuclei were stained with Hoechst (orange). Surface plot profiles (ImageJ) of the fluorescence intensity of rat IgGs measured in whole tumor cross-sections demonstrate the enhanced extravasation of IgGs in GLV-1h68-colonized tumors. The asterisk (a) indicate a significant difference between experimental groups (*** *P *< 0.001; Student's *t *test). All images are representative examples. Scale bars represent 500 μm (a), 300 μm (b), 1 mm (c, d).

The physiological response to infection and injury often includes vasodilatation, hyperpermeability, and neo-angiogenesis [27]. To investigate the effect on the tumor vasculature of long-term viral infection in the GI-101A breast tumor model, we measured the diameter of blood vessels in 42-days infected and uninfected control GI-101A tumors. The results, shown in Figure 4a, indicated that VACV infection (42 days p.i.) of the tumor tissue leads to a significant increase in vessel size (mean diameter 24.17 +/- 9.07 μm) in infected tumor areas compared to control tumors (10.09 +/- 4.01 μm).

Pericytes are another key component in vascular development, stabilization, maturation, and remodeling and are intimately associated with endothelial cells [28]. To analyze pericyte coverage in 42-days infected and uninfected control tumors, we compared α-SMA labelling in tumor cross-sections. Control tumors showed a large number of pericytes loosley attached to the tumor vasculature (Figure 4b). In contrast, however, VACV-infection of GI-101A tumors leads to regression and/or destruction of pericytes in the VACV-infected tumor tissue (Figure 4b).

Since the loss of pericytes may influence vessel permeability and tumor perfusion, we were interested to see whether the extravasation pattern of intraveneously injected Immunoglobulins (IgGs) differ in GLV-1h68-treated and untreated GI-101A tumors. Indeed, viral infection of the tumor tissue resulted in elevated extravasation of unspecific rat IgGs in colonized tumor areas (Figure 4c). Further, the comparison with control tumors revealed a heterogeneous intratumoral IgG extravasation pattern in control tumors (Figure 4c) and a locally restricted IgG extravasation pattern in GLV-1h68 infected tumor areas (Figure 4c), which indicated that the permeability of tumor vessels in infected tumor areas was strongly increased. Together, these results suggested that tumor colonization with GLV-1h68 activated the tumor endothelium which leads to increased tumor perfusion and leakage of blood-borne particles.

### Viral infection induces massive leukocytes recruitment via the activated tumor vasculature

**Figure 5:**
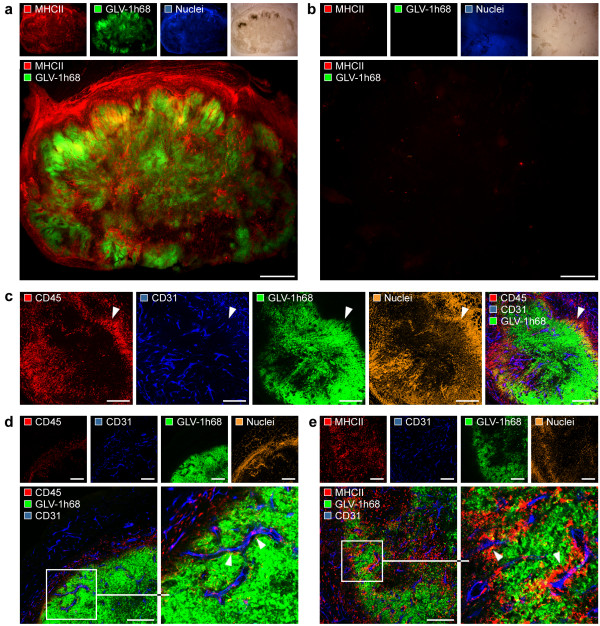
**Induced leukocyte recruitment via the activated tumor vasculature in GLV-1h68 infected tumors**. GI-101A tumor-bearing mice were i.v. injected with 5 × 10^6 ^pfu of GLV-1h68 and tumors were harvested 42 days p.i.. (a, b) Whole tumor cross-sections of GLV-1h68-infected tumors (a) and control GI-101A tumors (b) were labelled with anti-MHCII antibody to visualize the recruitment of MHCII-positive leukocytes. (c) Confocal image of GLV-1h68-colonized tumors revealed accumulation of CD45-positive leukocytes around viral patches. (d, e) Confocal images showed intense interaction of CD45-positive (d) and MHCII-positive leukocytes (e) with the CD31-positive tumor vasculature. Immune cells were labelled with anti-MHCII antibody (red; a, b, e) or anti-CD45 antibody (red; c, d); nuclei were visualized using Hoechst (blue in a, b; orange in c, d, e), tumor vasculature was labelled with anti-CD31 antibody (blue) and viral infection was indicated by GFP fluorescence (green). All images are representative examples. Scale bars represent 1 mm (a, b), 300 μm (c-e).

The activated endothelium is characterized by vascular hyperpermeability and increased expression of adhesion molecules, which facilitate perivascular inflammatory cell infiltration [29]. We demonstrated above that tumor colonization with the oncolytic GLV-1h68 strain lead to increased hyperpermeability as well as to the up-regulation of the adhesion molecule CD31, which is directly involved in the transmigration of leukocytes via the endothelial barrier. Therefore, we analyzed the extent of tumoral leukocytes recruitment in GLV-1h68-infected and control mice. Indeed, 42-days colonized GI-101A tumors revealed an intense intratumoral recruitment of MHCII-positive cells (monocytes/macrophages, dendritic cells and B cells) to tumor xenografts compared to uninfected control GI-101A tumors of same age (Figure 5a,b, Additional file 2: Figure 2a,b). Interestingly, we found that the extensive viral load in the tumor tissue seem to be the trigger for massive leukocytes recruitment. In comparison less infected GLV-1h68-infected tumors at earlier time points (21 days p.i.) showed only slight recruitment of leukocytes (Additional file 2: Figure 2c,d). In addition, we could also show specific recruitment of CD45-positive leukocytes (common leukocyte antigen) around viral patches indicating an immunological response against GLV-1h68-infected cells (Figure 5c). Detailed microscopic analysis showed that a large number of CD45-positive as well as MHCII-positive leukocytes were associated with the tumor vasculature, which may represent sites of actively ongoing leukocytes recruitment via the activated tumor vasculature (Figure 5d,e).

### Viral-mediated inflammation creates an "immunovascular memory" in colonized GI-101A tumors

**Figure 6:**
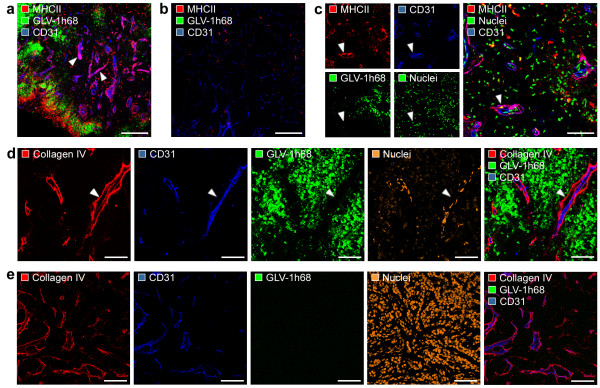
**Association of MHCII-positive leukocytes with the tumor vasculature**. GI-101A tumor-bearing mice were i.v. injected with 5 × 10^6 ^pfu GLV-1h68 and tumors were harvested 42 days p.i., prepared immunohistologically and examined using confocal microscopy. (a, b) Association of MHCII-positive leukocytes cell cluster with the CD31-positive tumor vasculature (arrowhead) in GLV-1h68-infected tumors (a); isolated MHCII-positive cells in control tumors (b). (c) MHCII^+^/CD31^+ ^cell invasion (arrowhead) of the endothelial intima and accumulation around tumor vessels. (d, e) The type IV collagen IV-containing basal membrane (red) of the tumor vasculature was thickened and loosley attached to endothelial cells (arrowhead) in GLV-1h68-infected tumors (d) compared to control tumors (e). MHCII-positive leukocytes were labelled with anti-MHCII antibody (red; a-c); tumor vasculature was marked with anti-CD31 antibody (blue), GFP fluorescence indicate viral-infected cells, and nuclei were stained with Hoechst (green in c; orange in d, e). All images are representative examples. Scale bars represent 300 μm (a, b), 75 μm (c-e).

It is already described for atherosclerosis that infectious agents may lead to enhanced accumulation of monocytes/macrophages at the endothelium which are surrounded by an increased deposition of extracellular matrix components (e.g. collagen). This vessel-associated accumulation of immune cells serve as an "immunovascular memory" which lead to an ever-growing immunological response by reiterative immune cell recruitment [30]. To test whether similar morphological alterations of the tumor vasculature may occur in response to tumor colonization with GLV-1h68, we analyzed the interaction of MHCII-positive leukocytes with the tumor vasculature in detail. Indeed, we could identify a close association of MHCII^+^/CD31^+ ^cells (vascular leukocytes) with the CD31-positive tumor vasculature, which is restricted to massively infected, late-stage (42 days p.i., Phase III) GLV-1h68-colonized GI-101A tumors (Figure 6a) and not detectable in control tumors (Figure 6b). Additional detailed confocal microscopic analysis revealed that the MHCII^+^/CD31^+ ^cells invaded the endothelial intima and accumulated around tumor vessels (Figure 6c).

Furthermore, the analysis of the type IV collagen-containing basal membranes of the tumor vasculature in 42-days colonized GI-101A tumors and uninfected control tumors, showed thickened collagen matrices around vessels in GLV-1h68-colonized tumors only (Figure 6d). Lastly, we could identify a space between CD31-positive endothelial cells and the thickened basal membrane only in viral-infected GI-101A tumors (Figure 6d,e).

These results strongly suggests that oncolytic treatment of human xenografts lead to the accumulation of MHCII-positive cells in late infection stages located between the endothelial lining and the subjacent, thickened basal lamina. This virus-induced formation of local atherosclerotic tumor vessels may serve as a potential "immunovascular memory".

### The role of leukocytes in the GLV-1h68-mediated tumor elimination

We have seen an enormous recruitment of MHCII-positive leukocytes in late infection stages (regression phase III) of GLV-1h68-infected GI-101A tumors, therefore, we set out to analyze the role of the leukocytes in the viral-mediated tumor elimination process.

**Figure 7:**
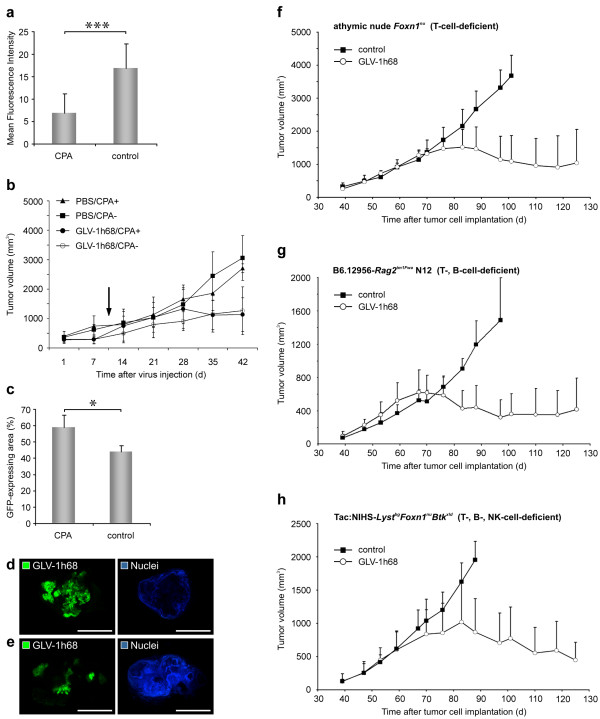
**MHCII-positive immune cells, T-, B-, and NK cells did not significantly contribute to VACV-mediated tumor regression**. GI-101A tumor-bearing mice were intraveneously (i.v.) injected with either 5 × 10^6 ^pfu/100 μl GLV-1h68 (a-e) or 1 × 10^6 ^pfu/100 μl GLV-1h68 (f-h) or PBS as a control. Immunosuppression was reached using 100/140 mg/kg cyclophosphamide monohydrate. (a) Fluorescence intensity measurement of recruited MHCII^+ ^leukocytes in GLV-1h68 infected GI-101A tumors treated or untreated with CPA. The fluorescence signal was measured in 4 images of each tumor (n = 5). Shown are the mean values +/- standard deviations. (b) Tumor growth was monitored weekly by measuring the tumor volume of five mice in each group. Shown are the mean values +/- standard deviations. The study was repeated in an independent experiment. CPA treatment was started 10 days p.i. (arrow). (c-e) Distribution of GLV-1h68 within the tumor tissue 42 days p.i. was visualized by GFP in whole tumor cross-sections of 5 different tumors; the corresponding 8-bit grey-scale images of GFP and nuclei were used for calculation of the extent (%) of viral infection in tumor cross-sections using ImageJ software. Shown are the mean values +/- standard deviations. (d, e) Representative images of CPA-treated GLV-1h68-infected tumor (d) compared to untreated GLV-1h68-infected tumor (e) revealed a higher degree of oncolytic tissue destruction. (f-h) Tumor growth in athymic nude *Foxn1*^*nu *^(f), B6.12956-*Rag2*^*tm1Fwa *^N12 (g), and Tac:NIHS-*Lyst*^*bg*^*Foxn1*^*nu*^*Btk*^*xld *^(h) mice was monitored weekly by measuring the tumor volume of four to five mice in each group. In all models mice were intraveneously (i.v.) injected with 1 × 10^6 ^pfu/100 μl GLV-1h68 at day 39 post tumor cell implantation. Shown are the mean values + standard deviations. The asterisk (a, c) indicate a significant difference between experimental groups (* *P *< 0.05, *** *P *< 0.001; Student's *t *test). Scale bars represent 5 mm.

To determine the contribution of recruited immune cells to rejection of virally infected GI-101A tumors, we treated infected and uninfected tumor-bearing nude mice with the broad-spectrum immunosuppressive agent CPA [19,31]. We started with CPA-treatment 10 days p.i. to establish substantial viral burden in all experimental groups, however, temporally still before massive infiltration of immune cells occurred (data not shown). The evaluation of immunosuppression by CPA in nude mouse models revealed that the recruitment of MHCII-positive cells in CPA-treated, VACV-infected GI-101A tumor-bearing mice significantly decreased in comparison to untreated, VACV-infected GI-101A tumor-bearing mice (Figure 7a). Furthermore, the histological examination of spleen sections showed also reduced MHCII-positive cell densities in the monocyte/macrophage-containing red pulp, whereas the B-cell-containing white pulp was not affected (data not shown). To determine whether the immune system (mainly MHCII-positive monocytic cells) was instrumental in or detrimental to oncolytic VACV efficacy, we evaluated the tumor growth pattern of uninfected and VACV-infected tumor-bearing animals either treated or untreated with CPA. In general, CPA treatment of uninfected GI-101A tumor-bearing mice did not *per se *significantly change the tumor growth pattern (Figure 7b). The CPA-treated, VACV-infected group showed no significant difference in the tumor growth characteristics compared to the untreated, VACV-infected group. However, the study also revealed that tumors of CPA-treated, VACV-infected animals showed earlier regression suggesting a more effective viral replication which may be due to the elimination of an anti-viral immune responses.

To investigate the effect of immunosuppression on viral replication and distribution within the tumor tissue, which determines the degree of viral-mediated tumor oncolysis, we measured the virus content in tumors using GFP-imaging and the content of viable tumor cells using Hoechst-labelling. The histological analysis of the viral distribution (equivalent to GFP) in CPA-treated and untreated animals revealed that the extent of the GFP-positive area in immunosuppressed tumors was significantly increased compared to untreated tumors (Figure 7c-e). Therefore, necrotic destruction of the tumor tissue in CPA-treated, VACV-infected animals is more pronounced than in untreated, infected animals as shown by decreased Hoechst-labelling of viable tumor cells.

To analyze the contribution of T-, B-, and NK-cells to the VACV-mediated tumor regression process, we analyzed human GI-101A tumor growth upon VACV infection in athymic nude *Foxn1*^*nu *^(T-cell-deficient), B6.12956-*Rag2*^*tm1Fwa *^N12 (T-, B-cell-deficient), and Tac:NIHS-*Lyst*^*bg*^*Foxn1*^*nu*^*Btk*^*xld *^(T-, B-, NK-cell-deficient) mouse strains. The tumor growth curves showed similar tumor growth and regression characteristics in all three mouse models (Figure 7f-h). Therefore, these immune cells did not contribute to VACV-activated anti-tumoral immune response and are not required for VACV-mediated tumor regression.

In summary, the results suggest that neither the MHCII-positive immune cells nor the T-, B-, or NK-cells are major contributors to VACV-mediated GI-101A tumor regression.

## Discussion

The mechanisms involved in tumor regression during oncolytic therapy are still a matter of heated debate. They may naturally vary among different tumor models and/or be dependent on different oncolytic virus strains used. Predominantly, oncolytic viruses are effective therapeutic agents due to their ability to efficiently infect and destroy cancer cells. In addition, replicating viruses may interfere with components of the tumor microenvironment such as the tumor vasculature and the immune system of the host. Therefore, oncolytic tumor destruction may be a multi-step process, in which the different components work with or against each other. In this study, we show that the oncolytic VACV GLV-1h68 drastically interfered with host components such as the tumor vasculature and induced also a massive innate immune response. But the predominant mechanism which leads to regression of tumors, in this model at late stage of tumor regression, was found to be direct viral-infection-mediated tumor cell destruction.

In contrast, Breitbach et al [32] showed that a significant portion of the tumor killing activity of vesicular stomatitis virus and VACV in the murine CT-26 colon cancer model is caused by indirect killing of uninfected tumor cells. In this tumor model the authors suggest that massive neutrophil activation followed by vascular damage and apoptosis of uninfected tumor cells one day after infection are the main cause of tumor cell destruction. However, the tumor killing may be caused by the high viral titer (1 × 10^9 ^pfu) used in the study to infect tumor-bearing animals. Further, the large amount of activated neutrophils may occlude the abnormal tortuous tumor vessels and the oxitative burst may directly destroy endothelial cells leading to vascular shutdown followed by apoptosis of surrounding tumor cells. In contrast we showed, that tissue necrosis exactly colocalizes with GFP-expressing VACV-infected tumor cells in human breast tumor xenografts. Interestingly, the infected, necrotic tumor areas remain highly vascularized in late infection stages (42 dpi). Therefore, neither vascular shutdown nor other indirect killing activities seem to occur and tumor destruction parallels with the site of viral replication and spreading.

Destruction of the tumor vasculature seems to be, in general, a promising strategy to induce tumor shrinkage by deprivation of nutrients and oxygen. In this regard, the oncolytic viruses itself may target, infect and destroy the tumor vasculature. For example, Kirn et al. [14] showed that an oncolytic vaccinia virus mutant strain with *B18R *deletion infects and destroys tumor-associated vascular endothelial cells. In contrast, we did not find any infected endothelial cells in tumors nor did the GLV-1h68 strain show general replication in murine or in human endothelial cell lines. On the other hand, combination of oncolytic viruses with anti-angiogenic therapy also appeared to enhance virotherapy, possibly due to the stabilization of the tumor vasculature or reducing the neovascular responses associated with viral replication [33-35]. Numerous therapy approaches are described using oncolytic viruses in combination with anti-angiogenic molecules to potentiate tumor destruction [11,12,34,36]. Beside the widely discussed starvation effects exhibited by angiogenesis-inhibitors, the destruction or normalization of the tumor vasculature may also have effect on the host immune response, due to a reduced infiltration of innate immune cells in the infected tumor tissue, which in turn affects virus survival.

In general, the vascular endothelium regulates innate and adaptive immune responses by controlling the extravasation of leukocytes from the blood into the inflamed tissue [27,29]. During inflammation, these patroling leukocytes extravasate into the tissue via a sequential process that is initiated by their adhesion to endothelial cells, which in turn leads to endothelial cell activation [29]. The activated endothelium is characterized by vascular hyperpermeability and increased tissue edema, which in turn facilitates perivascular inflammatory cell infiltration. The tumor vasculature in GLV-1h68-infected areas strongly resembles the activated endothelium in inflamed tissue, which is supported by the increased expression of CD31, the up-regulation of genes involved in leukocytes recruitment as well as the observed vasodilatation and hyperpermeability. However, the inflammatory response of the endothelium remains mainly localized in and directly around viral patches within the tumor tissue. At least, the encapsulation of viral patches by CD45- as well as MHCII-positive leukocytes indicated that the viral-induced immune response is mainly restricted to the invader and seems not to be a general anti-tumoral response in the late tumor regression phase.

Detailed confocal analysis of the tumor vasculature in late-stage infected tumors revealed a cell population, not yet described in the context of oncolytic tumor therapy, which coexpresses endothelial (CD31) and dendritic cell markers (CD45, MHCII) [37]. Recently, Conejo-Garcia et al. [37] described this novel leukocyte subset within ovarian carcinomas and attributed this cells to have the capacity to generate functional blood vessels in tumors. During oncolytic tumor therapy, this cell population may stabilze or partly restore the tumor vasculature within the infected, necrotic tumor areas, which represent truly unfavourable conditions for cell survival.

The observed phenomenon of vasodilatation as well as the increased permeability could also be enhanced by necrosis of the vessel-surrounding area, including destruction of pericytes, which reduces mechanical stress on the tumor vasculature leading to decompression of tumor vessels. Recently, Padera et al [38] showed, that tumor-specific cytotoxic therapy results in more efficient drug delivery by decompressing collapsed vessels. Therefore, we suppose that oncolytic VACV could be used as a "natural enhancer" of chemotherapy by improving the intratumoral dissemination of chemotherapeutics.

The relative importance of direct oncolysis versus immune-mediated tumor regression remains in most of the animal models uncertain. In general, xenografts are considered as chronically inflamed and do not by themselves provide sufficient signals to induce an acute inflammation leading to immune-mediated rejection of the tumor [39]. The generation of an effective anti-tumoral immune response depends on danger signals within the tumor, and infectious agents inherently offer these sufficient signals [40,41]. Therefore, viral infection of the tumor tissue may generate the essential trigger to alter the immune milieu of the tumor microenvironment. To elucidate whether the initiated immune response is directed against the viral invader or against the tumor tissue, we used the previously reported immunosuppressive agent CPA [19,31] to deplete virus-induced intratumoral immune cell recruitment. The tumor growth curve analysis showed no significant difference in the tumor growth characteristics between CPA-treated and CPA-untreated VACV-infected GI-101A tumors. However, we propose here that in general, factors such as viral distribution/degree of oncolysis or extent of tissue destruction/necrosis and correlation e.g. to the recruitment of immune cells offer much more information about therapy success than the tumor volume alone, because the tumor volume that was measured could be either necrotic debris or host cells of the tumor microenvironment instead of cancer cells. Our study showed that the extent of viral distribution/necrotic tissue destruction increased in CPA-treated, VACV-infected tumors and correlated with reduced intratumoral MHCII-positive immune cell infiltration compared to untreated, VACV-infected tumors. Although there is no significant change in the tumor growth characteristic upon immunosuppression, the significant higher extent of viral distribution/necrosis in these animals indicates that MHCII-positive immune cells impede viral-mediated tissue destruction. According to data shown here, immunosuppression increases oncolytic effects of a herpes simplex virus-derived OV [42] as well as an oncolytic adenovirus [19] via enhancing intratumoral viral spread. Further, Fulci et al [42] showed via clodronate liposome-dependent depletion of phagocytic cells, that mainly cells of the monocytic origin are responsible for clearance of intratumoral viral particles.

The immunolgical response against GLV-1h68, however, is not effective enough to eliminate the virus from the tumor tissue. This may be due to the wealth of immune evasion mechanisms presented by vaccinia virus [43]. Furthermore, the recruited immune cells may not as cytotoxic as usual due to the local immunosuppressing tumor microenvironment and can only slow down viral spread but not eliminate the viral infection focus. Alternatively, these recruited immune cells, which encapsulate viral patches, form an anatomical barrier, which may not be overcome by the virus due to their lesser susceptibility to VACV infection.

Previously, we have shown by transcriptional profiling of different tumor models that oncolytic GLV-1h68 infection does induce strong pro-inflammatory signatures [44,45]. Recently, Wang et al. [39] postulated that viral infection of the tumor tissue generates the necessary trigger that activates an acute immune response against the tumor tissue. However, the here described data revealed, that the immunological response in GLV-1h68-mediated GI-101A tumor destruction represents indeed an acute inflammation, but this response is only directed against the "pathogen" and not against the tumor tissue. We could show here that neither MHCII-positive immune cells nor T-, B-, or NK cells contribute significantly to VACV-mediated tumor regression. Collectively, these data support the primarily oncolytic character of VACV-mediated tumor destruction and demonstrate that the activation of a direct anti-tumoral immune response was not critical for tumor regression in this tumor model.

## Conclusions

In summary, the here presented results described the mechanism leading to oncolytic tumor therapy and highlighted the ongoing acute inflammation in the infected tumor tissue. The study revealed, supported by use of immunosuppressed animals, that the activated host response is directed against the invading virus and not against the tumor tissue. Therefore, we assume that targeted, local immunosuppression of tumors during oncolytic therapy should enhance VACV-mediated tumor tissue destruction and improve therapeutic outcome. Furthermore, the VACV-induced local inflammation within the tumor presents an ideal condition for therapeutic approaches, which combine oncolytic viruses with systemically injected chemotherapeutics due to the VACV-mediated enhanced tumor perfusion.

## Competing interests

This work was supported by grants from Genelux Corporation (R&D facility in San Diego, CA, USA). SW received a postdoctoral fellowship, VR and AW received a graduate fellowship by Genelux Corporation awarded to the University of Wuerzburg, Germany. YAY and AAS are salaried employees of Genelux Corporation and have personal financial interests in Genelux Corporation.

## Authors' contributions

SW conceived and designed the study, performed experiments, analyzed the data, and wrote the manuscript. VR helped to perform some experiments and analyzed data. YAY conceived, designed and carried out experiments with immunodeficient mouse strains, and helped to draft the paper. AW, EW, FMM conceived, designed and carried out the microarray analysis. AAS conceived the study, and participated in the design and coordination and helped to draft the manuscript.

All authors read and approved the final manuscript.

## Pre-publication history

The pre-publication history for this paper can be accessed here:

http://www.biomedcentral.com/1471-2407/11/68/prepub

## Supplementary Material

Additional file 1 **Specific oncolytic destruction of human GI-101A breast tumor xenografts**. GI-101A tumor-bearing mice were intraveneously (i.v.) injected with 5 × 10^6 ^pfu GLV-1h68. (a) Distribution of GLV-1h68 within the tumor tissue 42 days p.i. was visualized by GFP in whole tumor cross-sections of 6 different tumors; the corresponding 8-bit grey-scale images of GFP and nuclei were used for calculation of the extent (%) of viral infection in tumor cross-sections using ImageJ software. 55.25 +/- 7.26% of tumor cross-sections were colonized with GLV-1h68. (b) Whole tumor cross-sections (100 μm) of GLV-1h68-infected GI-101A tumors 7 and 21 days p.i. were stained with Phalloidin-TRITC (red) to label the actin cytoskeleton and Hoechst 33342 (blue) to visualize cellular nuclei; GFP fluorescence (green) indicated viral-infected cells. GLV-1h68-infection was restricted to small patches within the tumor tissue at early infection time points. (c) Infection of GI-101A-RFP tumors with GLV-1h68 (green) revealed specific infection of RFP-expressing GI-101A tumor cells (red) 42 days p.i.. The comparison of the RFP fluorescence intensity in 21- and 42-days-colonized tumors showed a decrease in the RFP signal 42 days p.i. demonstrating specific oncolytic tumor cell destruction. All images are representative examples. Scale bars represent 5 mm (a-c). Click here for file

Additional file 2 **Recruitment of leukocytes - massive intratumoral recruitment in 42-days-infected tumors and weak recruitment at earlier time points**. (a, b) 42-days-infected (left image in a, b) and control GI-101A tumors (right image in a, b) were labelled with anti-MHCII antibody (red) to visualize tumoral leukocyte recruitment. Confocal images showed peritumoral (a) and increased intratumoral (b) recruitment of MHCII-positive cells in GLV-1h68-infected tumors compared to control tumors; nuclei were visualized using Hoechst (blue); GLV-1h68-infected tumors showed GFP fluorescence (green). (c, d) 21-days-infected GI-101A tumors were labelled with anti-MHCII antibody (c) or anti-CD45 antibody (d). Early-infection stages of GI-101A tumors showed only mild, peritumoral recruitment of leukocytes. All images are representative examples. Scale bars represent 300 μm (a, b), (c) 2 mm. Click here for file
