# Supplementary figures and images for: Viral-mediated oncolysis is the most critical factor in the late-phase of the tumor regression process upon vaccinia virus infection
Source: BMC Cancer. 2011 Feb 14;11:68. doi: 10.1186/1471-2407-11-68 (PMC3044654; doi:10.1186/1471-2407-11-68)

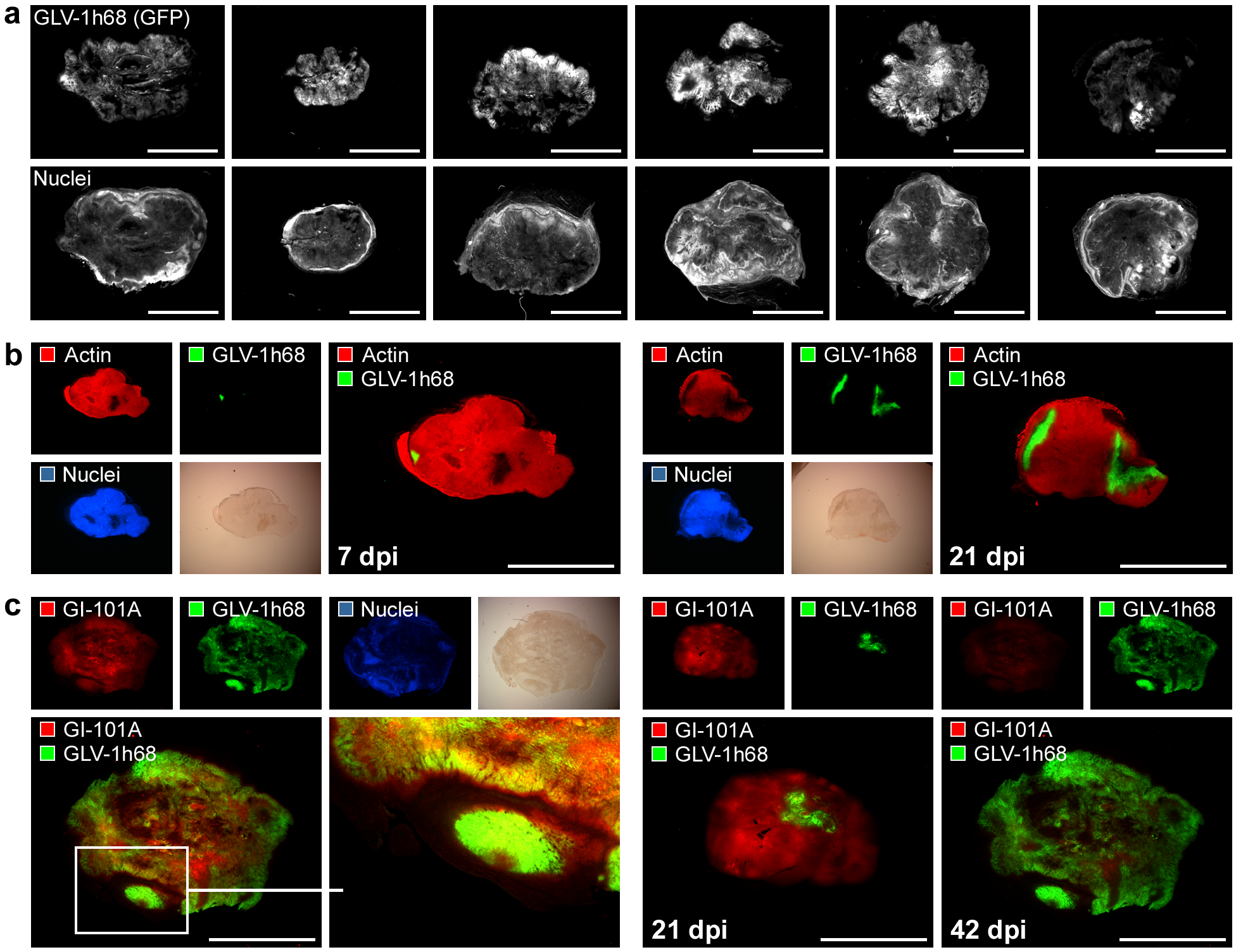

Supplement: Additional file 1 — Specific oncolytic destruction of human GI-101A breast tumor xenografts. GI-101A tumor-bearing mice were intraveneously (i.v.) injected with 5 × 10^6^ pfu GLV-1h68. (a) Distribution of GLV-1h68 within the tumor tissue 42 days p.i. was visualized by GFP in whole tumor cross-sections of 6 different tumors; the corresponding 8-bit grey-scale images of GFP and nuclei were used for calculation of the extent (%) of viral infection in tumor cross-sections using ImageJ software. 55.25 +/- 7.26% of tumor cross-sections were colonized with GLV-1h68. (b) Whole tumor cross-sections (100 μm) of GLV-1h68-infected GI-101A tumors 7 and 21 days p.i. were stained with Phalloidin-TRITC (red) to label the actin cytoskeleton and Hoechst 33342 (blue) to visualize cellular nuclei; GFP fluorescence (green) indicated viral-infected cells. GLV-1h68-infection was restricted to small patches within the tumor tissue at early infection time points. (c) Infection of GI-101A-RFP tumors with GLV-1h68 (green) revealed specific infection of RFP-expressing GI-101A tumor cells (red) 42 days p.i.. The comparison of the RFP fluorescence intensity in 21- and 42-days-colonized tumors showed a decrease in the RFP signal 42 days p.i. demonstrating specific oncolytic tumor cell destruction. All images are representative examples. Scale bars represent 5 mm (a-c). [file 1471-2407-11-68-S1.TIFF]

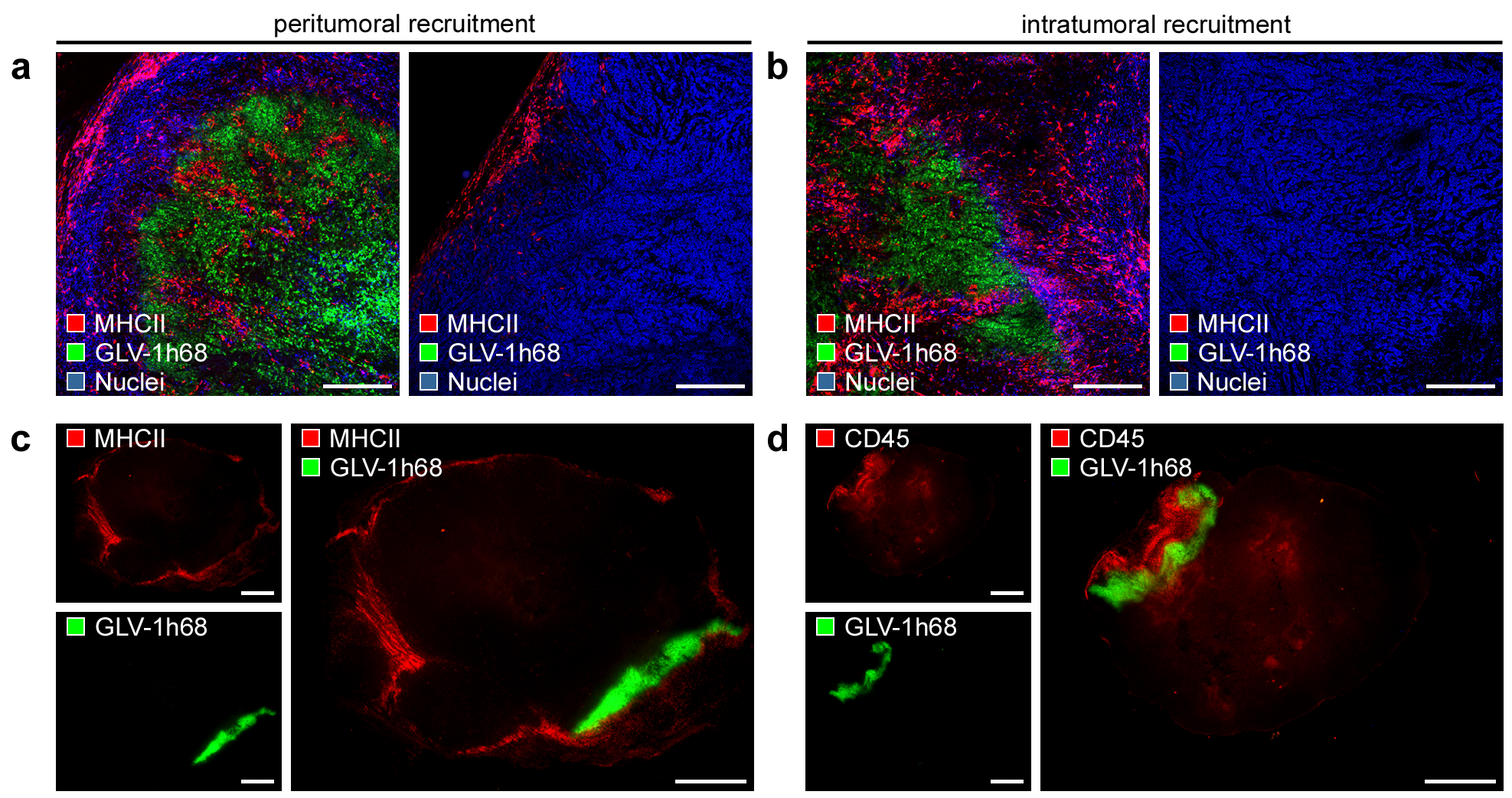

Supplement: Additional file 2 — Recruitment of leukocytes - massive intratumoral recruitment in 42-days-infected tumors and weak recruitment at earlier time points. (a, b) 42-days-infected (left image in a, b) and control GI-101A tumors (right image in a, b) were labelled with anti-MHCII antibody (red) to visualize tumoral leukocyte recruitment. Confocal images showed peritumoral (a) and increased intratumoral (b) recruitment of MHCII-positive cells in GLV-1h68-infected tumors compared to control tumors; nuclei were visualized using Hoechst (blue); GLV-1h68-infected tumors showed GFP fluorescence (green). (c, d) 21-days-infected GI-101A tumors were labelled with anti-MHCII antibody (c) or anti-CD45 antibody (d). Early-infection stages of GI-101A tumors showed only mild, peritumoral recruitment of leukocytes. All images are representative examples. Scale bars represent 300 μm (a, b), (c) 2 mm. [file 1471-2407-11-68-S2.TIFF]
